# Oxidant/antioxidant effects of chronic exposure to predator odor in prefrontal cortex, amygdala, and hypothalamus

**DOI:** 10.1007/s11010-015-2430-2

**Published:** 2015-05-16

**Authors:** G. E. Mejia-Carmona, K. L. Gosselink, G. Pérez-Ishiwara, A. Martínez-Martínez

**Affiliations:** Departamento de Ciencias Químico Biológicas, Instituto de Ciencias Biomédicas, Universidad Autónoma de Ciudad Juárez, Anillo envolvente del Pronaf y Estocolmo S/N, Zona Pronaf, C.P. 32315 Ciudad Juárez, Chihuahua Mexico; Department of Biological Sciences and Border Biomedical Research Center, University of Texas at El Paso, 500 W University Ave., El Paso, TX 79902 USA; Instituto Politécnico Nacional, Escuela Nacional de Medicina y Homeopatía, Guillermo Massieu Helguera 239, Fracc. La Escalera, Ticomán, C.P. 07320 Mexico, Distrito Federal Mexico

**Keywords:** Oxidative stress, Amygdala, Superoxide dismutase, Hypothalamus, Prefrontal cortex, Anxiogenic stress

## Abstract

The incidence of anxiety-related diseases is increasing these days, hence there is a need to understand the mechanisms that underlie its nature and consequences. It is known that limbic structures, mainly the prefrontal cortex and amygdala, are involved in the processing of anxiety, and that projections from prefrontal cortex and amygdala can induce activity of the hypothalamic–pituitary–adrenal axis with consequent cardiovascular changes, increase in oxygen consumption, and ROS production. The compensatory reaction can include increased antioxidant enzymes activities, overexpression of antioxidant enzymes, and genetic shifts that could include the activation of antioxidant genes. The main objective of this study was to evaluate the oxidant/antioxidant effect that chronic anxiogenic stress exposure can have in prefrontal cortex, amygdala, and hypothalamus by exposition to predator odor. Results showed (a) sensitization of the HPA axis response, (b) an enzymatic phase 1 and 2 antioxidant response to oxidative stress in amygdala, (c) an antioxidant stability without elevation of oxidative markers in prefrontal cortex, (d) an elevation in phase 1 antioxidant response in hypothalamus. Chronic exposure to predator odor has an impact in the metabolic REDOX state in amygdala, prefrontal cortex, and hypothalamus, with oxidative stress being prevalent in amygdala as this is the principal structure responsible for the management of anxiety.

## Introduction

Stress induces a neurochemical cascade that can cause long-lasting changes. Activation of the hypothalamic–pituitary–adrenal (HPA) axis is the most characteristic response to stress in mammals. In anxiety, the prefrontal cortex (PfC) sends projections and dopamine inputs to the amygdala [1] initiating a glucocorticoid cascade through the HPA axis [2]. Neuronal imaging studies in humans have shown that some anxiety and depressive disorder patients have increased amygdaline function [3]. The increase in neuronal activity in the amygdaline zone during anxiety periods produces cardiovascular changes mediated by excitation of neurons in the hypothalamus [4]. Predator odor induces a fear and anxiety-like behavior in rodents similar to some anxiety disorders in humans in which risk assessment, fight, flight, and exploration conducts are observed, that is why in the past decade this model has been used to evaluate the effects of fear and anxiety-related diseases such as general anxiety disorder (GAD), posttraumatic stress disorder (PTSD), and panic disorder and depression [5–8]. Predator odor stress induces an elevation in dopamine turnover in the medial prefrontal cortex (mPfC) and amygdala [9] and changes in afferent and efferent transmissions of the amygdala [10, 11]. It selectively activates diverse nuclei of the amygdala, PfC, and hypothalamus [12], structures that are rarely activated in immobilization, foot shock, or forced swimming models of stress [13]. Furthermore, predator odor stress induces cardiovascular, behavioral, endocrine, and autonomic long-term changes [4, 12].

Cardiovascular modifications have been associated to an increase in metabolism with oxygen consumption and production of ROS by electron transport chain [14], which can lead to oxidative stress. The compensatory response can include increased antioxidant enzymes activities, overexpression of antioxidant mRNAs of enzymes, and genetic shift that could include the activation of antioxidant genes [15, 16]. This effect has been observed by Arzate-Vázquez et al. [17], when a modification in transcript expression occurred in amygdala, hypothalamus, hippocampus, and PfC in rats exposed to a chronic live predator exposure paradigm; later, we observed modification in the activity of some antioxidant enzymes under an acute model of predator odor [18]. Both models include an anxiolytic component and have studied structures responsible for the processing of emotions. Nevertheless, the effect of chronic predator exposure as an anxiogenic stimuli on the cellular antioxidant mechanisms of the amygdala, PfC, and hypothalamus has been poorly explored. Thus the aim of this study was to determine the effect that chronic anxiogenic stress has on the oxidant/antioxidant capacity of amygdala and PfC centers for processing emotions, and hypothalamus, the primary structure involved in arising the stress response.

## Methods

### Animals and treatment

All experimental protocols were approved by the Bioethics Committee of the Universidad Autónoma de Ciudad Juárez, following the guidance of Official Mexican Standard NOM-062-ZOO-1999. Twelve single-housed, experimentally naïve, adult male *Sprague–Dawley* rats weighing 250–280 g (approximately 2 months of age) were randomly assigned to control or stress group. The model of chronic predator odor exposure was used according to Dielenberg et al. [19] with some modifications. Control group, without exposure to cat odor, was exposed to a 25 × 25 cm clean fabric. Stress group or cat odor-exposed group was exposed to a 25 × 25 cm fabric in which a domestic cat slept overnight followed by vigorous rubbing against the fur of the cat. Treatment cage for both groups consisted of two parts, as described by Dielenberg et al. [12]: two-thirds of the cage were made with clear transparent Plexiglas while one-third consisted of a black Plexiglas with a peaking hole, meant to be a hiding box. Treatment was applied for five consecutive days as follows: First, a habituation period of 20 min in the treatment cage, followed by 20 min in their housing cage and finally, 20 min in the treatment cage but with their respective fabrics (without cat odor for control or cat odor for stress) attached to the opposing wall from the black Plexiglas. On the 6th day, the rats were euthanized by decapitation for ROS evaluation or by intraperitoneal overdose of pentobarbital for enzyme activities and TBARS.

### Corticosterone measurement

Plasma corticosterone (CORT) was determined before and after predator odor exposure. Three days before treatment, blood from the saphenous vein was drawn to measure basal levels of CORT and measured again on control and stressed rats on the day of euthanasia (Day 6). After pentobarbital injection, blood was drawn from cardiac puncture, collected in 1 mg/mL EDTA- containing microtubes, centrifuged at 3000×*g* for 10 min, then plasma was separated, and stored at −80 °C. CORT concentration was measured using the AssayMax Corticosterone ELISA kit (Assaypro EC3001-1) following manufacturer’s instructions. Results are expressed as ng of CORT per mL of plasma.

### Preparation of tissue for enzyme activity and TBARS

Rats euthanized by overdose injection of pentobarbital were perfused for 20 min with ice-cold Krebs-Henseleit solution containing 148 mM NaCl, 4 mM KCl, 1.85 mM CaCl_2_, 1.05 mM MgCl_2_, 3 mM HEPES, 13.5 μM EDTA pH 8.0, 5.5 mM glucose, and 0.0035 % p/v ascorbic acid [20]. Whole brain was washed in ice-cold 0.9 % saline solution and frozen at −20 °C, then Amygdala, PfC, and hypothalamus were dissected and immersed in solution containing 50 mM cold sodium phosphate buffer of pH 7.4 and 1 % phenylmethylsulfonyl fluoride (PMSF) in a ratio of 1:10 w/v. Tissues were homogenized and sonicated 3 times at 30 w (6.9 kHz; Sonic Dismembrator, Model 100, Fischer Scientific) for 10 s at 1-min intervals on ice. Homogenates were centrifuged at 20,800×*g* for 30 min at 0 °C [21]; the supernatants were kept at −80 °C until use to evaluate superoxide dismutase (SOD), glutathione S-transferase (GST), and thiobarbituric reactive substances (TBARS) levels.

### Superoxide dismutase

SOD activity was determined by auto-oxidation of pyrogallol (Sigma, Cat. No. 254002). The pyrogallol method developed by Marklund and Marklund [22] is a convenient and reliable technique to measure the activity of SOD and it has been extensively used [23–27]. Detection of SOD by pyrogallol depends on the inhibition of pyrogallol auto-oxidation by the enzyme, which can reach up to 99 % [22]. The specific detection of mitochondrial SOD, which is dependent on manganese (MnSOD), relies on the addition of sodium cyanide (NaCN) to inhibit cytosolic SOD, that is in turn dependent on copper and zinc (Cu/ZnSOD), thereby facilitating the detection of MnSOD [22]. Thus, a modified reaction of the method described by Marklund and Marklund [22] was used. A microplate reaction mixture containing 270 μL of 50 mM Tris–HCl pH 8.2 containing 1 mM diethylenetriaminepentaacetic acid (DTPA), 2.7 μL of 100 mM NaCN and 14 μL of supernatant was prepared. Reaction was started by the addition of 16 μL of 3.6 mM pyrogallol. MnSOD activity was determined in a microplate reader; inhibition of pyrogallol auto-oxidation was registered at 15-s intervals for 12 min and 2 s of mixing at 420 nm and room temperature. Only the first-order reaction at initial maximal velocity (V_o_ = V_max_; [S]_i_ >>> 10 km; [S]_i_ ≥ 0.9[S]_f_) was taken into account (at *r* = 0.99). One unit of MnSOD activity (U) was defined as the amount of enzyme that inhibits pyrogallol autoxidation rate by 50 % per min. It is expressed as U per mg of protein.

### Glutathione S-transferase

The activity of GST was determined according to the method of Habig et al. [28] with some modifications. On the day of the assay, equimolar substrate solutions of 2.25 mM 2,4-Dinitrochlorobenzene (CDNB, Sigma Cat. No. 237329) and 2.25 mM GSH (Sigma Cat. No. G-4251) were prepared separately in 0.1 M phosphate buffer of pH 6.5. Equal volumes of each substrate solution (2.25 mM CDNB and 2.25 mM GSH) were mixed and heated to 37 °C. Assay was prepared by adding 25 μL of supernatant to a 96-well plate followed by 200 μL of the substrate solution mix to start the reaction. Formation of the GS-CDNB complex was registered for 22 min at 15-s intervals for 5 s of mixing in a microplate reader at 340 nm and 37 °C. Only the first-order reaction at initial maximal velocity (V_o_ = V_max_, [S]_i_ >>> 10 km; [S]_f_ ≥ 0.9[S]_i_) was taken into account (at *r* = 0.99). One unit of GST activity (U) was defined as the amount of enzyme that catalyzes the formation of one µmol of GS-CDNB complex per min. We expressed GST activity as mU per mg of protein.

### TBARS

To determine TBARS, 100 µL of supernatant was mixed with 750 µL of 20 % acetic acid of pH 3.5, 750 µL of 0.8 % tio-barbituric acid (TBA), and 100 µL of 0.8 % sodium dodecyl sulfate (SDS). Reaction tube was incubated in a water bath at 90 °C for 90 min, then a sample of 200 μL of the reaction was taken, and measured at 532 nm in a microplate reader [29]. TBARS equivalents were calculated as the nmol of Malondialdehyde (MDA) per mg of protein calculated with the slope of a standard curve of 1,1,3,3-tetramethoxypropane (TMP).

### ROS equivalents

Because the rapid decay of ROS makes them unsuitable to be followed by the method used for antioxidant enzymes and TBARS, a fast frozen method was followed [30]. Rats were decapitated, the head was dropped and covered with ice, then the brain was dissected and frozen in dry ice; thereafter, amygdala, PfC, and hypothalamus were dissected and collected in ice-cold Locke’s buffer containing 154 mM NaCl, 5.6 mM KCl, 3.6 mM NaHCO_3_, 2.0 mM CaCl_2_, 10 mM d-glucose, 5 mM HEPES of pH 7.4, at a concentration of 50 mg/mL [30]; tissues were homogenized and then sonicated once at 30 w (6.9 kHz, Sonic Dismembrator, Model 100, Fisher Scientific) for 10 s on ice. Homogenates were then centrifuged at 54×*g* for 20 min at 0 °C; the supernatant was kept and diluted to 1:10 ratio in ice-cold Locke’s buffer. ROS equivalents were measured with a modified method of Driver et al. [30] as follows: initially, a 9.18 mM of 2′,7′-dichlorfluorescein (DCFH) solution was prepared by mixing 0.6 mg of DCFH diacetate (DCFH-DA, Sigma Cat. No. D6883) with 6 μL of 1 M NaOH, incubated for 10 min, and then 3.6 μL of 1 N HCl was added, followed by 124.4 μL of absolute ethanol [31]. Then the solution was diluted up to 367 µM working solution in Locke’s buffer. Reaction mixture consisted of 260 μL of diluted supernatant and 40 μL of 367 μM DCFH. Absorbance was measured at 502 nm in a microplate reader each minute for 60 min at room temperature. ROS equivalents were calculated from the slope of a H_2_O_2_ standard curve, divided by the mg of protein to be expressed as pmol of ROS per min and mg of protein.

### Statistical analysis

Comparisons were made between the control and stress groups for all variables; the data are expressed as the mean ± standard deviation (SEM for graphs) for six rats per group, or nine in the CORT assay. Significance was determined by Student’s *t* test at *p < 0.05, or **p < 0.005.

## Results

**Fig. 1:**
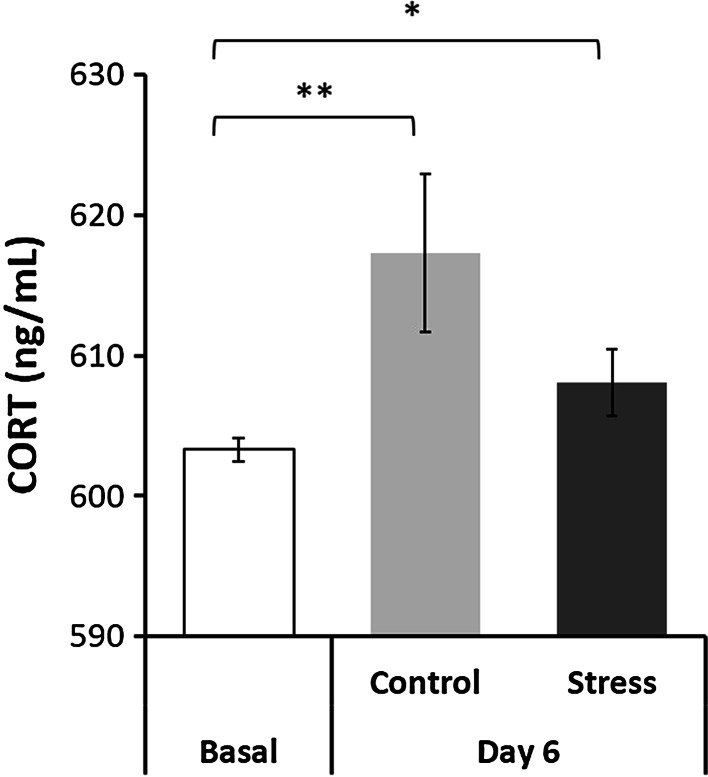
Plasma corticosterone in response to stress. Basal represents concentration of CORT (ng per mL) 3 days before treatment (*n* = 18, all rats). Day 6 represent values for control (*n* = 9) or stressed groups (*n* = 9). Mean values ± SEM. *t* test significance at *p < 0.05, **p < 0.005 compared to basal

**Fig. 2:**
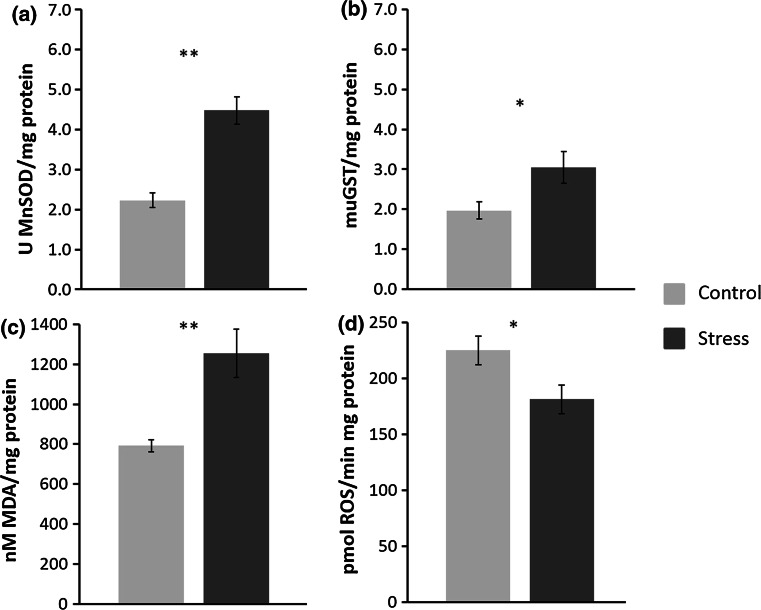
Antioxidant capacity of Amygdala. **a** MnSOD activity, results are expressed as U of MnSOD per mg of protein. **b** GST activity, results are expressed as mU of GST per mg of protein. **c** TBARS, results are expressed as nM of MDA per mg of protein. Bars represent mean values (*n* = 6) ± SEM. **d** ROS equivalents, results are expressed as pmol of ROS per mg of protein. *t* test significance at *p < 0.05; **p < 0.005 control versus stress (*n* = 6 for each condition)

Overall results of the oxidant/antioxidant effect of chronic exposure to predator odor indicate that chronic exposure to predator odor increases plasma corticosterone level (Fig. 1) as expected in stressed mammals. In amygdala, chronic stress induces oxidative stress as seen by the increase in TBARS (Fig. 2c); this oxidative stress increases antioxidant protection provided by enzymes such as MnSOD (Fig. 2a) and GST (Fig. 2b); these events seem to maintain the homeostasis in amygdala since the ROS equivalents are counterbalanced (Fig. 2d). While in PfC and hypothalamus, chronic stress increases the antioxidant protection as seen by the increase in MnSOD but neither TBARS or ROS levels were changed (Table 1).

**Table 1:** Biochemical markers of oxidative stress in prefrontal cortex and hypothalamus

	Group	Hypothalamus	Prefrontal cortex
MnSOD	Control	3.03 ± 0.87	4.00 ± 0.98
	Stress	4.19 ± 0.67*	5.93 ± 0.44**
GST	Control	4.11 ± 0.87	5.36 ± 0.59
	Stress	4.75 ± 0.42	5.77 ± 0.52
TBARS	Control	341.30 ± 66.96	787.70 ± 118.17
	Stress	401.30 ± 95.88	558.00 ± 207.64*
ROS	Control	164.83 ± 26.22	186.52 ± 61.55
	Stress	160.4 ± 26.00	245.68 ± 18.79

Control (rats not exposed to cat odor), Stress (rats exposed to cat odor). Mitochondrial superoxide dismutase (MnSOD) expressed as U per mg of protein. Glutathione S-transferase (GST) expressed as mU per mg of protein. Thiobarbituric acid reactive substances (TBARS) expressed as nM of malondialdehyde (MDA) per mg of protein. Reactive oxygen species (ROS) expressed as pmol per mg of protein. Results expressed as mean ± standard deviation. *T* test at * p < 0.05, ** p < 0.005 compared to control group. *n* = 6 for each condition

### Corticosterone

CORT was measured before and after chronic exposure to predator odor. After exposure to cat odor, a statistically significant increase in CORT was seen in both groups. CORT level was found to be increased to a greater extent in the control group (617.3 ± 16.9 vs 603.3 ± 4.0, *p* < 0.005)when compared to that in the stress group (608.1 ± 7.0 vs 603.3 ± 4.0, *p* < 0.05) and that at basal (Fig. 1).

### Antioxidant capacity

To evaluate the effect of chronic predator odor exposure on the antioxidant capacity, MnSOD and GST were measured. Mitochondrial SOD (MnSOD) activities in amygdala, PfC, and hypothalamus were measured using the pyrogallol method. As result of chronic exposure to predator odor, MnSOD activity had a tendency to increase in all tissues. Amygdala underwent the most radical change, showing increased MnSOD activity which is of two-fold (2.23 ± 0.40 vs 4.48 ± 0.77, *p* < 0.005; Fig. 2a); MnSOD activity increased in PfC by 50 % (4.00 ± 0.98 vs 5.93 ± 0.44, *p* < 0.005; Table 1) and increased by almost 40 % in hypothalamus(3.03 ± 0.87 vs 4.19 ± 0.67, *p* < 0.05, Table 1). Specific activity of GST was evaluated by the rate of formation of the complex GS-CDNB. In Amygdala of stressed rats, it had a 38 % statistically significant increment (1.97 ± 0.21 vs 3.05 ± 0.96; *p* < 0.05; Fig. 2b). No significant change of GST activity was observed in PfC and hypothalamus (Table 1). These results indicate that chronic exposure to predator odor induces the antioxidant enzyme response in amygdala and specific response of MnSOD in PfC and hypothalamus.

### Oxidative capability

The oxidant effect that chronic predator odor exposure has in amygdala, PfC, and hypothalamus was measured with TBARS and ROS equivalents. TBARS equivalents measured as the amount of MDA produced showed a considerable 150 % increase in amygdala from stressed rats compared to control group (793 ± 61 vs 1253 ± 242, *p* < 0.005; Fig. 2c). However, contrary to what happened in amygdala, a significant decrease in lipid peroxidation by 40 % was observed in PfC (787 ± 118 vs 558 ± 207, *p* < 0.05, Table 1). Interestingly, hypothalamus showed a statistically non-significant trend with increase in MDA products (Table 1). ROS equivalents measured as the production of oxidized DCFH showed a statistically significant decrease of 20 % in amygdala (225 ± 28 vs 181 ± 29, *p* < 0.05; Fig. 2d). While PfC had a 31 % non-significant increasing trend in ROS levels (Table 1), hypothalamus of stress group rats had a non-significant 3 % decreasing trend in ROS levels (Table 1). Therefore results suggest that oxidative stress is present in amygdala while an antioxidant protection is seen in PfC.

## Discussion

Previous work has shown that different stress models can induce the antioxidant response and oxidative stress in diverse tissues and brain structures [21, 32–35]. To our knowledge, none has explored the effects that anxiogenic stress, such as predator odor, have on the oxidant/antioxidant balance. Previous work by our group evaluated the antioxidant response to an acute exposure to predator odor [18]. Nevertheless, anxiety is often described as a prolonged feeling of uncertainty to a stressor [36], rather than a sudden acute stimulus. Therefore, we evaluated the oxidative and antioxidant responses of structures responsible for handling emotional stress in a chronic model of predator odor exposure. The antioxidant enzymes activities of MnSOD, GST, as well as lipid peroxidation (TBARS) and ROS equivalents were measured in amygdala, PfC, and hypothalamus of rats exposed to predator odor for five consecutive days. The most statistically significant results were observed for amygdala, with an increase in antioxidant enzymes and TBARS and a relatively mild decrease in ROS. On the other hand, in PfC an increment in MnSOD activity was related to a decrease of TBARS. And finally, only a subtle increase in MnSOD was seen in hypothalamus.

### Corticosterone response to anxiogenic stress

CORT has been widely used as an indicator of stress; nevertheless, variation in CORT response has been found depending on the type and duration of the stressor [37]; most agree that one stressful event triggers a more intense response by HPA axis than a chronic stress [32, 38, 39]. In a model of chronic unpredictable stress and reduced ambiance temperature [40], CORT levels decrease compared to that in non-stressed animals. Figuereido et al. [41] used a model of acute and chronic exposure to predator odor and they found that response of HPA axis to repetition of the same stressor is sensitized, and even more, introduction of new environment and manipulation displayed an elevation in CORT levels greater than the one seen with the stressor. We measured CORT before and after 5 days of 20-min exposure to predator odor. Our results showed that CORT remained elevated in cat odor-exposed rats even after exposition had ended; this response indicates that during the 5-day treatment, elevation of CORT has occurred suggesting sensitization of the HPA axis. On the other hand, control group showed a greater, non-significant elevation in CORT than the cat odor-exposed group at day of euthanasia. Given that the control group had not been exposed to a stressful experience until the time of euthanasia, elevation of CORT is considered normal due to manipulation and restraint at the time of injection of pentobarbital [42]. File et al. [43], in a model of acute and chronic predator odor, found that a single exposure to cat odor induces activation of HPA axis, in contrast with a repetitive 5-day exposure paradigm in which CORT response is equal to that elicited by a neutral non-threatening odor, but greater than basal. Setting aside the effect exerted by manipulation during euthanasia, File’s group results are in concordance with ours.

### Oxidative stress in the amygdala

The amygdala acts as a convergence zone in response to emotional stressors, thus the importance of understanding its oxidative status relies on its capability to maintain an adequate transfer of information. The status of antioxidant enzymes has been poorly assessed in amygdala. In a model of chronic lidocaine administration, Cano-Europa et al. [44] reported a modification in the REDOX environment in amygdala and changes in oxidative markers during the initial administration of lidocaine. Using the chronic mild stressor protocol, Abelaira et al. [45] reported no change in lipid peroxidation and SOD. Here, we have shown that chronic exposure to predator stress can induce enzymatic antioxidant response in amygdala by elevating activities of MnSOD and GST, while decreasing the amount of ROS (Fig. 2). Elevation in MnSOD activity suggests that production of superoxide by electron transport chain increased, and thus increasing enzyme activity. Activity of GST has been linked to the defense against organic hydroperoxides and oxidative stress [46]. The increase in GST activity and rise in TBARS in response to chronic exposure to predator odor could mean that oxidative stress is present within the amygdala (Fig. 2), and although phase I and II antioxidant enzymes increased, the REDOX homeostasis in the cell is disrupted resulting in the elevation of lipid peroxides (Fig. 2) and consequently oxidative stress. The observed decrease in ROS (Fig. 2) could be due to the elevation of antioxidant enzymes, but it is also consistent with the increment in TBARS which is an indicative of the reaction between ROS and lipids to form lipid peroxides [47]. Previously we published the enzymatic antioxidant response to acute anxiogenic stress (a single exposition to predator odor for 1 hour) in which a tendency to increase MnSOD and GST was found although being not statistically significant [18]. Thus, our previous work suggests that acute stress does not induce an antioxidant response in amygdala, but the work presented in this paper shows that anxiogenic chronic stress does.

### Antioxidant capacity of prefrontal cortex

Although PfC is a crucial structure involved in stress processing, few studies have been published evaluating the impact that stress has in its REDOX state. Different groups have investigated the influence of chronic stress in the development of oxidative stress but with diverse results. For instance, Zlatković et al. [32] found that chronic social isolation compromises the activity of SOD and increases lipid peroxidation. The same group, Filipović et al. [39] found a trend of increased protein expression of MnSOD in a chronic isolation model. Furthermore, in a chronic administration of d-amphetamine, activities of SOD and GST had no change while in a chronic mild stressor protocol a decrease in SOD activity and increase of MDA products was found [45, 48]. Our results showed that chronic exposure to an anxiogenic stimuli induces an antioxidant protection in PfC by elevation of MnSOD activity and decrease of lipid peroxides. Increase in MnSOD activity would suggest that an elevation in production of superoxide occurred during chronic stress, then conversion of hydrogen peroxide to hydroxyl radical would be expected to be followed by elevation in lipid peroxides [49, 50] leading to oxidative stress. Nevertheless, a decrease in lipid peroxidation was seen in chronically stressed animals suggesting that H_2_O_2_ scavenging mechanisms are in place as no change in ROS and a protective effect from MnSOD were observed.

### Antioxidant activity in the hypothalamus

Previous studies have consistently measured the activity of hypothalamus in response to stressors by means of corticosterone [51–53], but, few importance has been given to the integrity of its cells, even less to its oxidant/antioxidant balance. For instance, Djurasevic et al. [54] evaluated the enzymatic antioxidant response of ascorbate supplementation, finding a decrease in ROS and Cu/ZnSOD but no change in MnSOD. Chronic administration of d-amphetamine does not change SOD activity but increases GST [55] while an acute emotional stressor decreased activity of SOD [56]. The hypothalamus is the main structure activated by most stressors and its antioxidant capacity has not been thoroughly investigated. Therefore we evaluated the oxidant/antioxidant status of the hypothalamus following chronic predator odor exposure. Hypothalamus from stressed animals had an increase in activity of MnSOD, but no changes in ROS or lipid peroxides. Enzymatic activities and oxidative stress markers were measured 1 day after finalization of treatment. According to previous HPA axis activation analysis via quantitation of corticosterone in plasma [43], increased activity in the hypothalamus should be seen at the first days of exposition to predator odor. Thus, the fact that at day of euthanasia only MnSOD activity was altered means that there was an increase in metabolic activity in the hypothalamus, and that what we are seeing is the remaining of the antioxidant response that occurred earlier in the experiment. Activation of MnSOD suggests that hypothalamus had an increase in production of superoxide radical presumably via respiratory chain [49], which can in turn trigger transcription of the MnSOD gene rising the enzyme level and its activity [57]. Thus hypothalamus showed a protective antioxidant effect in response to a chronic anxiogenic exposure.

Amygdala and PfC in collaboration with hippocampus are considered the main structures to control activity of the HPA axis. Specifically amygdala and PfC are mostly implicated in the consolidation and modulation of the hypothalamic stress response to psychological stimuli [58, 59]. We suggest that the oxidant/antioxidant capability of amygdala might be permanently altered as it was the most affected by the chronic anxiogenic stress, while PfC is resilient to the stressor. In the case of hypothalamus, as only activity of MnSOD activity was slightly affected we can suggest that feedback mechanisms involving amygdala and PfC act to control hypothalamic response and sensitize the HPA axis to prevent oxidant/antioxidant imbalance. Furthermore, as mentioned before, the HPA axis response can vary depending on the type, duration and repetition of the stressor, displaying an intense response at the beginning of the stress and sensitizing when the stress becomes chronic. Therefore, indicators as behavior, biochemical, neuronal, and oxidative markers should also be measured to assess the biological state of stress and anxiety of mammals.

## Conclusions

It has been shown that stressful events that may cause anxiety lead to oxidative stress in various tissues or even trigger the onset of neurodegenerative diseases and cancer [47, 60]. We evaluated the effect that chronic predator odor exposure has on the oxidant/antioxidant capacity of amygdala, PfC, and hypothalamus. Our results showed an increased activity of MnSOD in all studied structures, indicating a specific activation mechanism of MnSOD in response to chronic anxiogenic stress. The notorious alteration in the REDOX state of Amygdala, suggests that it is the most sensitive structure to the effect of chronic anxiogenic stress. All oxidative markers measured were modified in amygdala, indicative of oxidative stress. While in plasma a sensitization of the HPA axis developed as seen by CORT. To our knowledge, this is the first study to investigate the effect of chronic predator odor in the oxidant/antioxidant capability of amygdala, PfC, and hypothalamus. Although much is left to explore in the antioxidant mechanisms involved in response to predator odor and other anxiogenic stressor, we propose that oxidative markers can be useful to evaluate the biological state of stress of mammals. Then, future work should include a temporal analysis of oxidative markers, in plasma and limbic structures, by increasing the days of exposure and evaluation of the effect of anxiogenic stress in transcript and protein expression of antioxidant enzymes and antioxidant-related transcription factors.
